# A Trust-Based Model for Secure Routing against RPL Attacks in Internet of Things

**DOI:** 10.3390/s22187052

**Published:** 2022-09-17

**Authors:** Syeda Mariam Muzammal, Raja Kumar Murugesan, Noor Zaman Jhanjhi, Mamoona Humayun, Ashraf Osman Ibrahim, Abdelzahir Abdelmaboud

**Affiliations:** 1 University Institute of Information Technology, PMAS Arid Agriculture University, Rawalpindi 46000, Pakistan; 2 School of Computer Science, SCE, Taylor’s University Lakeside Campus, Subang Jaya 47500, Malaysia; 3 Department of Information Systems, College of Computer and Information Sciences, Jouf University, Sakaka 72388, Saudi Arabia; 4 Faculty of Computing and Informatics, University Malaysia Sabah, Kota Kinabalu 88400, Malaysia; 5 Department of Information Systems, King Khalid University, Muhayel Aseer 61913, Saudi Arabia

**Keywords:** internet of things, IoT security, trust, RPL attacks, routing attacks, rank, blackhole

## Abstract

In IoT networks, the de facto Routing Protocol for Low Power and Lossy Networks (RPL) is vulnerable to various attacks. Routing attacks in RPL-based IoT are becoming critical with the increase in the number of IoT applications and devices globally. To address routing attacks in RPL-based IoT, several security solutions have been proposed in literature, such as machine learning techniques, intrusion detection systems, and trust-based approaches. Studies show that trust-based security for IoT is feasible due to its simple integration and resource-constrained nature of smart devices. Existing trust-based solutions have insufficient consideration of nodes’ mobility and are not evaluated for dynamic scenarios to satisfy the requirements of smart applications. This research work addresses the Rank and Blackhole attacks in RPL considering the static as well as mobile nodes in IoT. The proposed Security, Mobility, and Trust-based model (SMTrust) relies on carefully chosen trust factors and metrics, including mobility-based metrics. The evaluation of the proposed model through simulation experiments shows that SMTrust performs better than the existing trust-based methods for securing RPL. The improvisation in terms of topology stability is 46%, reduction in packet loss rate is 45%, and 35% increase in throughput, with only 2.3% increase in average power consumption.

## 1. Introduction

The Internet of Things (IoT) is a network of smart objects connected with each other and the Internet. In the IoT, smart devices exchange information and process data. IoT devices and applications are increasing exponentially. However, there are certain hurdles in adopting IoT by end-users, specifically the security and privacy concerns. In sensitive applications, for example, smart healthcare, data security becomes a critical matter for end-users and service providers [1,2]. The substantial exchange of data is incredibly crucial in IoT networks, and it is prone to breaching attacks. Due to inadequate security solutions in IoT, a number of disruptive attacks have been reported in recent years [3]. In addition, the expansion and immense growth of IoT will increase the security risks, threats, and the impact of attacks in the future [4,5].

**Figure 1:**
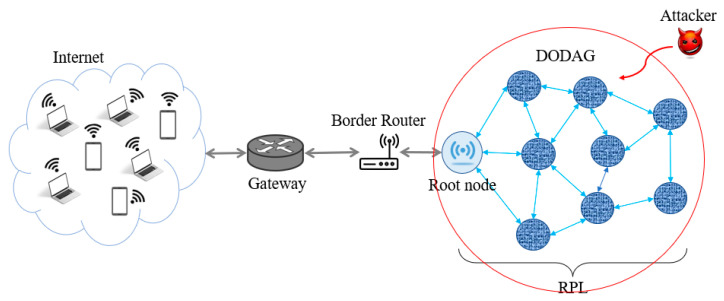
RPL-based Network in IoT.

IPv6 Routing Protocol for Low Power and Lossy Networks (RPL) is introduced by the Internet Engineering Task Force (IETF) to efficiently solve the routing needs in IoT [6]. Like other protocols, however, a considerable number of routing attacks are possible in RPL [7,8] which include RPL-specific attacks and the attacks inherited from Wireless Sensor Networks (WSNs) because both IoT and WSNs domains are related to each other [9]. Security from routing attacks is challenging in IoT networks because of the specifications and requirements of IoT systems and devices. Provided the prevalent applications of RPL for a smart world [10], it is imperious to deal with related security attacks [11,12]. Figure 1 presents the overview of RPL network, functional flow, and illusion of intrusion by an attacker in an RPL Destination Oriented Directed Acyclic Graph (DODAG). Some of the attacks in RPL include, Rank attack, Blackhole attack, Version number attack, Sinkhole attack, Wormhole attack, and DODAG Inconsistency attack.

In the existing literature, various methods for solving the IoT routing security are proposed, including intrusion detection systems, machine learning, and trust-based techniques [13]. Because of its simple deployment and incorporation into the IoT network, the trust-based approach is a potential way to fulfil RPL security needs. Several trust-based solutions have been proposed by researchers, specifically for the security of RPL-based IoT. In existing trust-based security solutions, node mobility is not at all considered [14,15] or is inadequate for mobile sink nodes [16], in RPL-based IoT for defense against routing attacks. The Rank and Blackhole attacks in RPL are the most disruptive routing attacks, among others. In a Rank attack, false rank information is advertised by the malicious node. As a result, the malicious node is selected as a potential parent. Similarly, the occurrence of Blackhole attacks in routing causes the dropping of packets and data loss.

Trust is a term defined for affiliation between two entities, where one entity is the evaluator, known as trustor, while the other is being evaluated, known as trustee [17]. In a trust relationship, the trustor must have confidence in the trustee regarding belief, benevolence, and honesty. Therefore, the trust value must make sure that the trustee would not betray the trustor by performing any malign actions [18]. The notion of trust is being used in various disciplines, including sociology as well as computer science, particularly, communications, networks, and the IoT [19].

The behaviour, integrity, and reliability of a sensor node are termed as trust in sensor networks in the domain of computer science. In the network and communications security, trust is a relation between participating entities. Trust relationship relies on past experiences and current circumstances of entities in the network and determines the efficiency, reconfigurability, and scalability. Certain metrics are used for a node’s cumulative trust value estimation, which reflects its legitimacy in the network. The node’s indirect or direct neighbours use this trust value to engage this node in network topology and route creation. Trust evolves over time with the changing trust metrics.

This research work intends to focus specifically on the security of the RPL routing protocol in the IoT. A trust-based model is proposed to improve security in RPL-based IoT, against Rank and Blackhole attacks. The trust-based approach is applied for the detection and isolation of malicious nodes. Trust metrics are selected through critical analysis and investigation of their suitability to enhance security for RPL in a mobile IoT environment [20]. Moreover, the proposed model, named SMTrust, considers the mobility metrics for trust computation. SMTrust is evaluated considering static nodes, mobile sender nodes together with a mobile sink node. SMTrust routing algorithms are embedded into RPL, and the protocol is assessed in terms of network performance, including topology stability, throughput, packet loss rate, and power consumption.

The performance of the proposed SMTrust model is evaluated via simulation using ContikiOS/COOJA simulator. The simulation results demonstrate that the performance of SMTrust is better as compared to standard RPL and existing trust-based methods, for instance, SecTrust [14], DCTM [16], and MRTS [15]. Following is the summary of contributions of this research work-at hand.

- We intend to analyze and adapt trust metrics, including but not limited to, the node’s behavior, characteristics, and mobility, in a bid to secure the RPL routing protocol.
- We intend to improve the algorithms for trust computation and trustworthy parent selection for attack detection.
- We intend to implement the preliminary SMTrust model, proposed in [21], by integrating it in the standard RPL routing protocol.
- We intend to evaluate the proposed model via simulation, and parameters such as, topology stability, packet loss rate, throughput, and power consumption to determine its performance as compared to the existing methods.

Though the number of IoT devices and applications is increasing exponentially, the security aspects are one of the factors hindering its widespread adoption. This research specifically will help in enhancing the IoT networks and routing security, and generally will facilitate the widescale adoption of the IoT. This research paper is structured as follows. Section 2 describes the motivation behind this research work and reviews the existing secure routing protocols. Section 3 explains the proposed SMTrust model along with the process flow, proposed parent selection algorithm, attack detection, and the computational complexity. Section 4 presents the experimental setup. Section 5 presents the simulation results and analyses the effectiveness as compared to the existing methods. Section 6 and Section 7 provide the discussion, conclusion, including future work and directions, respectively.

## 2. Related Work

### 2.1. Motivation

The most important component of the IoT is the networking that facilitates communication and interconnectivity. Particularly, routing holds a prominent place in networking, which involves building traffic routes for transmitting a packet from source to destination. Moreover, the security issue is crucial in networks, particularly routing, when the billions of devices are connected with each other, and the number of devices is predicted to be exponentially increasing in the coming years, as mentioned in a report by Statista [4].

Network security becomes challenging when a packet is routed through heterogeneous networks from resource-constrained devices to a server over the Internet. The IoT is a hybrid network that involves a number of heterogeneous networks, thus requires security solutions against network intrusions and disruptions [22]. In the IoT applications, there are different devices and computers connected with each other and use different operating systems and protocols. Hence, with the widespread IoT applications involving routing via RPL [23], it is imperative to address the related attacks. Out of several security solutions proposed for secure routing, a trust-based approach possesses the significance and viability for IoT networks and routing. A robust security solution will not only enhance protection against attacks but also facilitate the overall widescale adoption of IoT applications.

### 2.2. State-of-the-Art

IoT security has been worked upon continuously with the advent of smart devices. Numerous security techniques and solutions, including machine learning/deep learning [24], IDS-based, and trust-based [25,26,27,28] are proposed for IoT routing and network security. For addressing RPL attacks, existing mitigation techniques are typically based on either combining procedures in RPL or modifications in current RPL, for example, revising Objective Function (OF).

A trust-based method, SecTrust [14], is proposed for secure communication in IoT. By specifying its own OF, the proposed method is incorporated with RPL, thus establishing a new trust-aware and secure RPL routing protocol. Network efficiency and packet loss rate are assessed, including Sybil and Rank attacks detection. Trust evaluation, based on fuzzy logic, allows only trusted nodes to communicate through the threshold-based advertisement of trust with each other. Although their work in detecting and isolating attacker nodes is promising, its success against colluding attacks is not analysed. In addition, some of their conventions are relevant only to the static scenarios, such as smart home, and do not involve mobility-based parameters in trust computation or analysis. 

Similarly, a SecTrust revision is proposed [29] for the evaluation of Blackhole attacks using testbed. Moreover, the results are evaluated only for static topology. Furthermore, mobile nodes, power usage, and End-to-End delay are not analysed. For the IoT, a trust model based on multiple dimensions is proposed by [16] in which Quality of Service (QoS), Quality of P2P Communication (QPC), and contextual knowledge are incorporated. The model is embedded into RPL, and for Sybil, Blackhole, and Rank attacks, the results are evaluated. In contrast to default RPL objective functions, such as OF0 and MRHOF, the results show major improvements for average energy consumption, packet loss ratio, E2E delays, and parent change frequency. However, for practical situations involving attacks in mobile environments, the assessment is not sufficient. In comparison, mobile sink and its effects are not studied. Moreover, the authors’ focus is neither on how their model and trust metrics calculations are integrated into RPL nor how the attacks are detected with embedding trust [15].

In [30], a trust-aware model is proposed based on Random Forest and subjective logic for identification of sinkhole attack in RPL. Another trust-based IDS for RPL is proposed in [31] for solving the fabricated parent change vulnerabilities in RPL. Similarly, a trust-based authentication scheme is proposed [32] for mitigating the Rank, Sybil, Blackhole, and Man-in-the-Middle attacks. Ref. [33] presented a lightweight countermeasure against DODAG Information Solicitation (DIS) attack based on adaptively adjusted thresholds. Similarly, ref. [34] presented a load balancing mechanism to mitigate DIS flooding attacks. Ref. [35] introduced a new attack in RPL named Dropped Destination Advertisement Object (DDAO) and a distributed lightweight IDS to counter this attack. Ref. [36] proposed an Echelon value-based metric for objective function for early detection and isolation of Rank attack.

For social IoT, ref. [37] proposed trust-based and optimized RPL-based routing. Similarly, ref. [38] enhanced RPL for candidate parent nodes for mitigating worst parent attack in RPL. A reputation-based RPL protocol is proposed by [39] for detection of Selective Forwarding attack in IoT. A trust-aware and cooperative routing protocol, MRTS, is proposed by [15]. Trust calculation in MRTS is based on adding a new parameter, ETX. Their proposed method is effective in terms of packet delivery ratio, energy, throughput, and node rank change. MRTS uses the IDS approach for attacks detection and isolation. Hence from computational aspect, it requires a hardware security chip embedded in each node [40]. Furthermore, the mobility-based metrics and scenarios are not considered for testing its functionalities under routing attacks.

Table 1 presents the summary of recent literature related to routing attacks in IoT. In the existing research works, a number of trust models have been proposed for secure routing in IoT. They lack some features, such as consideration of IoT node mobility, heterogeneity in IoT environments, adaptability to IoT networks and routing, and consideration of RPL-specific attacks. Furthermore, trust dynamics and network performance are not taken into account in some of the presented solutions. However, some of the papers focus solely on network performance and routing behavior, neglecting to address routing attacks and security concerns. Moreover, critical security attacks, particularly routing attacks in RPL, are not evaluated in some trust-based network security solutions.

Traditional security solutions are insufficient to provide substantial security for smart applications. The use of trust models in IoT networks can reduce the uncertainty factor for nodes interconnection. The trust models proposed for IoT lack some critical features, including heterogeneity, dynamicity, and resource-constrained nature of IoT [43]. Some trust models involve computationally expensive IDS-based approaches for attacks detection, which require a hardware security chip embedded in the device [15]. As a result, a comprehensive trust model needs to be developed that considers the attacks with high impact such as Blackhole and Rank attacks, node mobility in an IoT environment, and the selection of appropriate trust metrics for an effective security solution for RPL-based IoT networks.

Our proposed model differs from the above-mentioned secure routing protocols in the selection of metrics, particularly involving mobility-based trust metrics along with direct and recommended trust. In terms of trust metrics, most of the existing methods use success rate, historical observations, and feedback which is also referred to as recommended trust. In addition to these metrics, SMTrust also employs the energy level, mobility, and the location and link stability. Table 2 shows the summary of trust metrics as employed by existing trust-based models. Secondly, in SMTrust, the malicious nodes are detected and isolated using a trust evaluation mechanism based on simple trust metrics calculations that do not require extra hardware to be embedded for security module, as in [15]. Additionally, SMTrust is evaluated for three different scenarios, based on static nodes, mobile sender nodes, and mobile sink node. To the best of our knowledge, only one study, DCTM [16], considers the mobile sender nodes, and there is no study in the literature that considers sink node mobility in an IoT environment.

Hence, SMTrust is proposed and evaluated for static and mobile nodes, including mobile sink node. The proposed protocol is proved to outperform in terms of topology stability, throughput, packet loss rate, and power consumption, comparing with recent trust-based routing protocols, for example, SecTrust [14], MRTS [15], and DCTM [16]. From the literature review, the types of routing attacks considered by recent research works, and the high impacts of Rank and Blackhole attacks, among others, in RPL, justify their selection for this research study.

## 3. Proposed SMTrust Model

A trust model is based on some trust factors. The main purpose of a trust-based model plays a significant role in considering particular trust factors to design an effective solution. In this research, a trust model is designed for providing security in RPL, and the trust factors are considered accordingly. The trust factors form up the quantifiable trust metrics. For the proposed SMTrust, the mobility of nodes is considered, which constitutes the mobility-based trust metrics. 

The proposed design of the SMTrust model for the security of RPL mainly consists of two phases: trust formation and attack detection. The trust formation includes trust metrics identification, trust metrics calculation, trust index computation, trust rating, and trust monitoring. The second phase includes attack detection and isolation of malicious nodes. A preliminary model and workflow of SMTrust is explained in [21].

### 3.1. System Model

**Figure 2:**
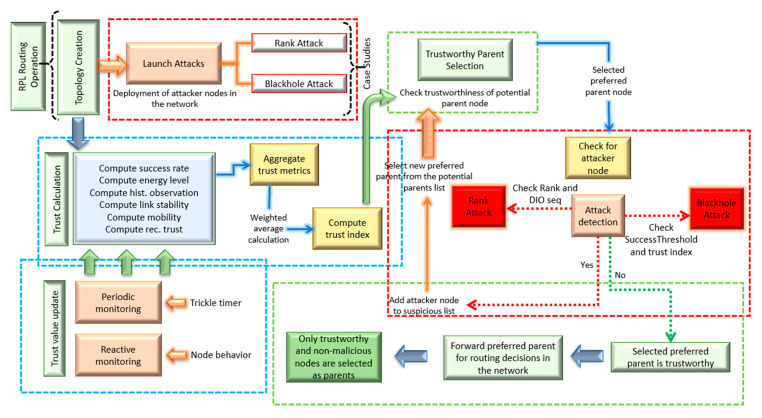
System Model of SMTrust.

Figure 2 depicts the system model of SMTrust including different phases and the flow of how trust propagates throughout the RPL operation. The trust formation phase and attack detection phase has been further divided into sub-phases. Each block of SMTrust system model is discussed below along with the processes involved.

#### 3.1.1. Topology Creation and Deployment of Attacks

SMTrust follows the RPL specification RFC 6550 for initialization of the normal routing operation. Similarly, the topology is created according to the default functioning and features of RPL, which is referred to as topology creation in proposed SMTrust model (Figure 2). The attacker nodes are deployed in the network for the considered case studies of Rank and Blackhole attacks. The particular attacker nodes are assumed to be positioned randomly among the legitimate nodes. The attacker process is launched by programming the nodes to act according to the attack type. For the Rank attacker nodes, the node is programmed to advertise the lowest rank to attract the traffic. Whereas for the Blackhole attacker nodes, the nodes are programmed to drop all the received packets.

#### 3.1.2. Trust Metrics Identification and Trust Index Calculation

The choice of suitable trust parameters is fundamental to the development of a trust-based security mechanism. The trust metrics employed by SMTrust, to provide IoT networks and routing security are scrutinized in detail [20]. For SMTrust, the most relevant trust metrics are considered, including historical observations, energy level, direct trust, and recommended trust. SMTrust incorporates mobility of nodes by considering mobility-based metrics in trust computation. Table 3 shows the description of applied trust metrics in SMTrust.

Using the calculations of trust metrics, the trust index is computed. The trust value computed is kept dynamic by assigning the appropriate weights (*w*) to the calculation of each trust metric. Hence, the trust index is the weighted aggregation of the trust metrics. The aggregated trust values are ranging from 0 to 1. The concept of a fuzzy threshold-based mechanism is used by SMTrust for the evaluation of trust rating [50]. The final trust index value is calculated using Equation (1).

*TrustIndex* = *w*_1_(*TM*_*SR*_) + *w*_2_(*TM*(*H*_0_)) + *w*_3_(*TM*_*EL*_) + *w*_4_(*TM*_*LLS*_) + *w*_5_(*TM*_*Mobility*_) + *w*_6_(*TM*_*RT*_) (1)

where (*w*_1_ + *w*_2_ + *w*_3_ + *w*_4_ + *w*_5_ + *w*_6_) = 1

Current or direct trust values are given more weightage than the historical observations. The degree of trust index, as evaluated by fuzzy judgement, is five-tuple, defined as, *T = [t1, t2, t3, t4, t5],* with the following trust levels, respectively, *[“No Trust”, “Poor Trust”, “Fair Trust”, “Good Trust”, “Full Trust”.* The defined trust levels are according to the given ranges of trust values as [0.0–0.20, 0.21–0.45, 0.46–0.70, 0.71–0.90, 0.91–1.00]. SMTrust does not consider the nodes that lie in *t1* and *t2* tuple for routing decisions and secure communication. The nodes in tuple *t4* and *t5* are considered as reliable and trustworthy to be forwarded for routing decisions. However, the nodes included in *t3* are in the middle of *“No Trust”* and *“Complete Trust”*. Hence, SMTrust tends to consider the nodes in *t3* only when there is a shortage or no nodes available in the range of *t4* and *t5* tuple for communication, following the strict calculation of the trust index. This is also to overcome the non-availability of nodes for routing. Hence, the trust threshold is kept at 0.46.

#### 3.1.3. Attack Detection

A preferred parent node is selected from the potential parents list and is checked for the attacker node using the attack detection procedure. For Blackhole attack detection, the node is checked for the success threshold and the trust index. Whereas for the Rank attack detection, the node is checked for the rank and DIO_seq as compared to the neighboring nodes. If the selected preferred parent is detected as an attacker node, it is added to the suspicious list and a new parent is required to be selected from the list of potential parents list using the parent selection procedure.

#### 3.1.4. Trustworthy Nodes Forwarding for Routing

If the selected preferred parent node is not detected as an attacker node, then it is forwarded as a trustworthy parent node for routing decisions in the network. Since the child node can send packets only to the selected parent node, and the parent nodes in SMTrust are verified to be trustworthy using the trust metrics computation and trust threshold for the parent selection procedure. Hence, only the legitimate nodes will take part in the network.

#### 3.1.5. Trust Value Update

A trust value needs to be updated regularly, that is, after particular time intervals. To update trust after regular intervals, SMTrust employs the trickle timer functionality in RPL, referred to as periodic trust update. The trickle timer is basically a timing mechanism used for optimizing the broadcast of DIO messages for preserving nodes’ resources. Similarly, another mechanism is adopted from RPL routing operation for trust value update, which is according to the node behavior change, referred to as reactive update of trust value.

The reactive update takes place when the behavior of the node changes and new parent needs to be selected from potential parents list. For instance, when the rank of the parent node changes, the child node has to align itself in the topology and hence tends to change its rank, as well as it needs to recompute the trust of the parent node according to its new position and behavior. Hence, the child node follows the trust computation, trustworthy parent selection, and attack detection process again to maintain a trustworthy topology.

### 3.2. Flow Diagram

**Figure 3:**
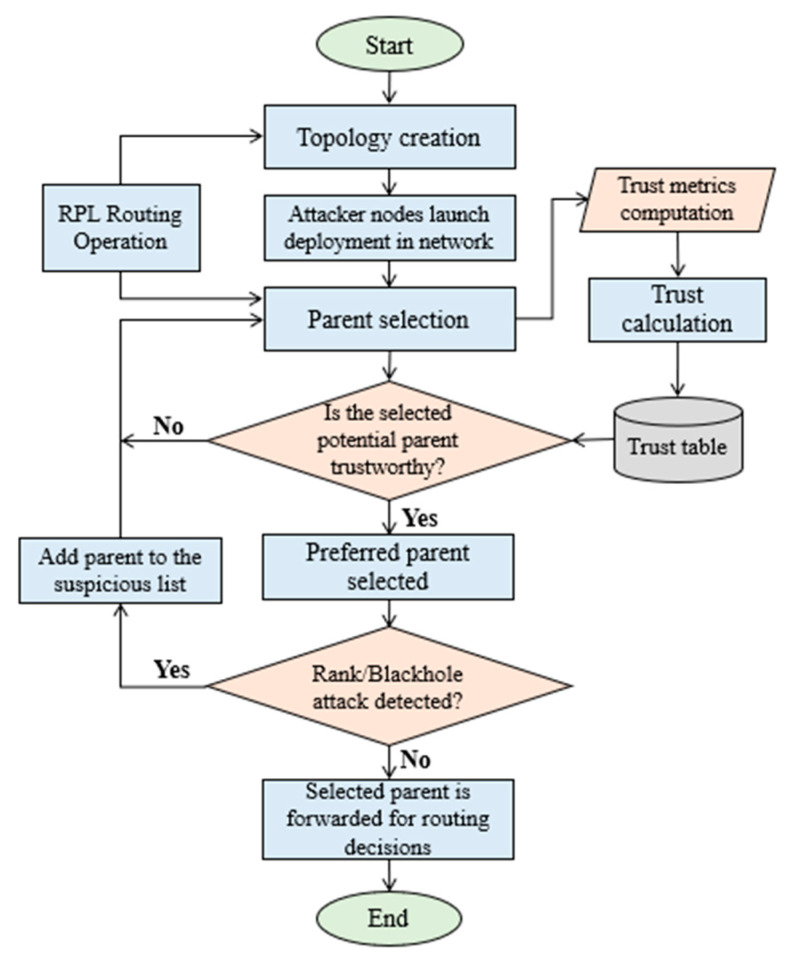
Process Flow of SMTrust.

The process flow of SMTrust is illustrated in Figure 3. After the RPL initialization steps and topology creation along with the deployment of attacker nodes in the network, a node is required to select a parent for itself in order to forward the packets. A list of potential parents is established, and trust is computed and stored in trust table for all the neighbouring nodes.

In the parent selection procedure, the potential parent is checked for trustworthiness. If the potential parent is trustworthy according to the set threshold value, then it is selected as the preferred parent. Otherwise, another node from the potential parent list is checked for the selection of preferred parent. Once the preferred parent node is selected, it is checked for malicious node. If the node is detected as an attacker, it is put in the suspicious list and another parent needs to be selected. If the node is detected as a normal and trustworthy node, it is forwarded for routing in the network.

### 3.3. Proposed Parent Selection Algorithm

The parent selection procedure of standard RPL is modified to develop a trust-based communication across DODAG. The proposed algorithm for trustworthy parent selection is illustrated below.

#### 3.3.1. Trust Computation in RPL

Trust computation assumes that all nodes are reliable in the beginning depending on the ICMPv6 and DIO packet exchange. As ContikiRPL assumes that nodes overhear neighbour nodes and their packet transmission [6], SMTrust also assumes the same. When RPL is initialized, preferred parents and routing decisions are determined, based on the specification in OF for trustworthy parent selection. Rank is computed for the nodes, according to the normal operations of RPL [51]. Once the topology is constructed and neighbour list is established, the trust values are computed according to the quantification of trust metrics, and the trust index is calculated by (1). Algorithm 1 illustrate the procedure for trust computation and parent selection facilitating the isolation of malicious nodes.
**Algorithm 1.** Trustworthy parent selection
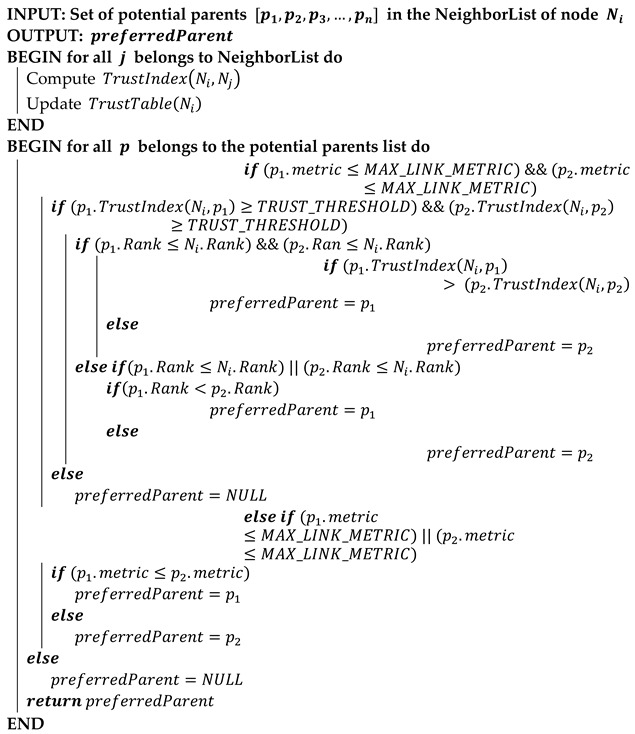


When a node receives DIO messages, the information communicated is used for routing table updates. The node calculates the trust values for its neighbors via the SMTrust trust computation mechanism. A set of trusted potential parents is then selected for an optimal path towards the root node. The selection of a trustworthy preferred parent ensures traffic routing reliability in the network. Finally, a new DIO message is generated and broadcasted to its neighbours, which contains the computed trust values, via DAG metric container. This method is followed by all the neighbouring nodes for the construction of DODAG. Afterward, the DODAG maintenance relies on the Trickle timer [6], limiting the transmission of control messages.

Once the trust index is calculated, it is not static, and rather keeps on updating considering two conditions, periodic and reactive monitoring. In SMTrust, a trickle timer algorithm for sending DIO messages in RPL is adopted for periodic update of the trust value. Whereas for reactive trust update, the monitoring process is initiated considering the changes in the behaviour of the node, for example, changing of rank without changing the DIO-seq (Rank attack). However, the trust value cannot be frequently updated, as it may affect the protocol’s performance, communication overhead, and network resources, such as energy, memory, and CPU cycles of a node. Hence, SMTrust tends to adopt the routing table update implementation in existing RPL as a foundation for the trust value update. Currently, SMTrust does not consider the recuperation of isolated nodes, which are classified as malicious or suspicious. This integration has been left as future work.

#### 3.3.2. Computational Complexity

For SMTrustOF, the objective function of standard RPL is modified, basically for trust-based parent selection and attacks detection. The complexity of trustworthy parent selection algorithm, Algorithm 1, is O(1) in the best case except the trust calculation for neighbouring nodes required as input for parent selection, which is O(n). Moreover, there can be n number of potential parents for a particular node, so in order to check the trustworthiness of the potential parents one by one, the complexity of Algorithm 1 is O(n) in the worst case. Similarly, the computational complexity for attack detection is O(1) in the best case, and O(n) in the worst case. Therefore, it can be deduced that the overall complexity for the added procedures in SMTrustOF is O(n). For SMTrust, the computational complexity increases with the calculation of trust metrics and aggregation.

#### 3.3.3. Rank and Blackhole Attacks Detection and Isolation of Attacker Nodes

In a Rank attack, the actual rank value is changed by the malicious node to advertise a better route for attracting traffic. However, the nodes which use this malicious node for routing their traffic end up losing the packets one way or the other. In the RPL network, a rank change occurs when a child node selects a new parent with a better rank. In a Rank attack, malicious nodes advertise themselves with fake ranks and optimal routes to their neighbours, which tend to select it as a new parent. The victim nodes select the new parent with less rank to obey the RPL rank rules for a loop-free topology. This results in the separation of these nodes from effective communication in the network.

**Figure 4:**
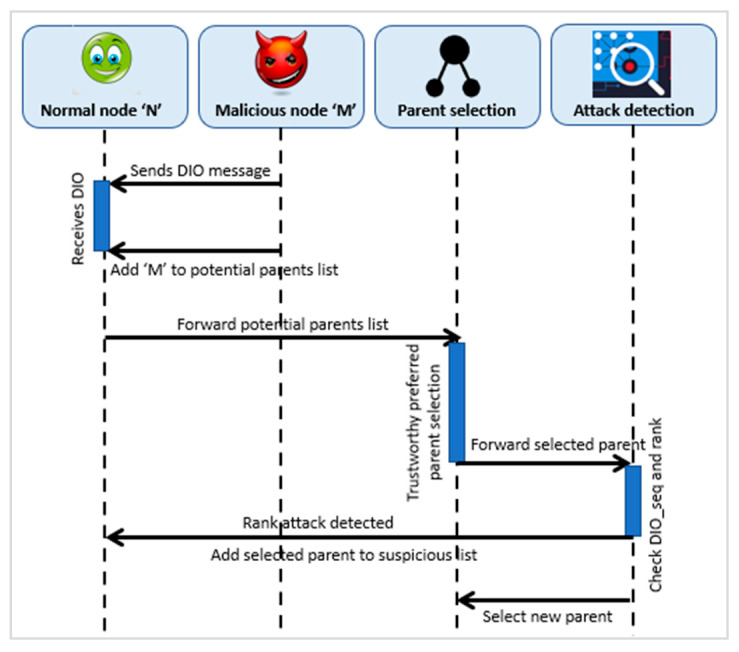
Detection of Rank Attack.

SMTrust employs the overhearing and monitoring of neighboring nodes to detect a Rank attack. Rank attack has several variants, including decreased rank attack, increased rank attack, and worst parent attack. This research work considers decreased rank attack. When DIO message is received by a node from a neighboring node, it checks if the rank changes without DIO_seq, that is, new DIO_seq is less than or equal to current DIO_seq, it means that it is a suspicious or fake DIO [52]; therefore, a Rank attack is identified. Moreover, checking the inconsistency of the rank of the potential parent against the neighboring nodes indicates a fake DIO and Rank value [14,52]. The sequence diagram for Rank attack detection is depicted in Figure 4.

**Figure 5:**
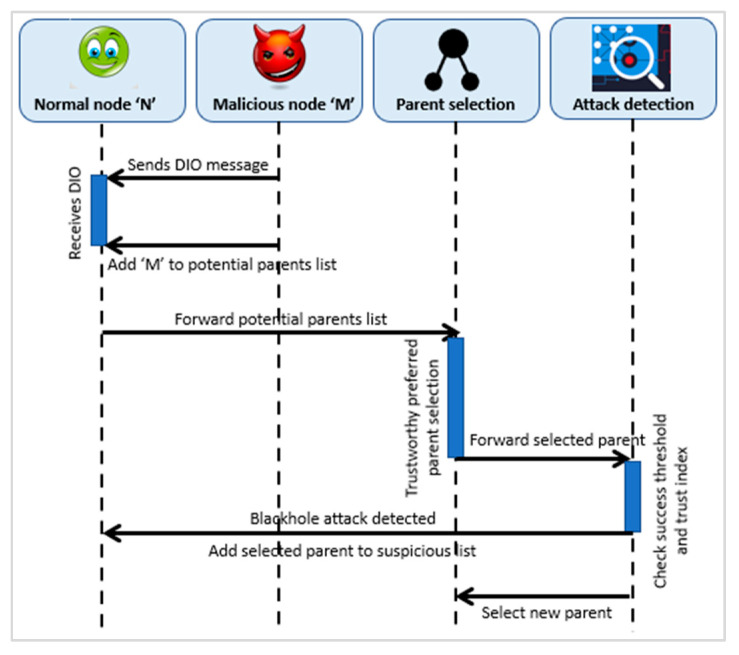
Detection of Blackhole Attack.

Similarly, a Blackhole attack is one kind of the packet dropping attacks. SMTrust accomplishes this by overhearing and monitoring the neighboring nodes whether the packets are being forwarded successfully or dropped by the trustee node. Blackhole attack is detected by the trust index of the preferred parent node. Moreover, an additional check is of success rate, to know that the packets are dropped by the node, and to confirm a packet dropping attack. Figure 5 depicts the sequence diagram of Blackhole attack detection.

According to the definition and detection of Rank and Blackhole attack, SMTrust adopts the concept of attack detection and isolation from [14,52].

## 4. Experimental Setup

To depict a real-world use case scenario, this research study replicates the deployment of IoT sensors in a smart hospital. Due to significant and critical assets at stake, including patients’ life, network security is a key issue for smart hospitals. There are several networked medical devices enabling remote patient monitoring. Stationary as well as mobile devices are used a lot in the smart hospital context. These devices include mobile devices, wearable external devices, stationary, implantable, and supportive devices [53].

Two types of network architectures can be considered for the smart hospital application: centralized and distributed. This research considers the centralized architecture where each node communicates with a sink or root node, also referred to as a controlling node. This type of setting is well supported by Contiki/Cooja simulator for a simple evaluation, according to the acknowledgment that exhaustive evaluation of the scenario will be too daunting to undertake. SMTrust carefully analyses and selects the simulation parameters values as adopted by [14,15,16] with justification for the assumptions suitable according to the real-world scenario. Table 4 shows the parameters setting in the Cooja simulator. Attacker nodes are positioned randomly among the normal nodes to illustrate a real use-case scenario where attacker nodes can infiltrate the internal network of the smart environment.

**Figure 6:**
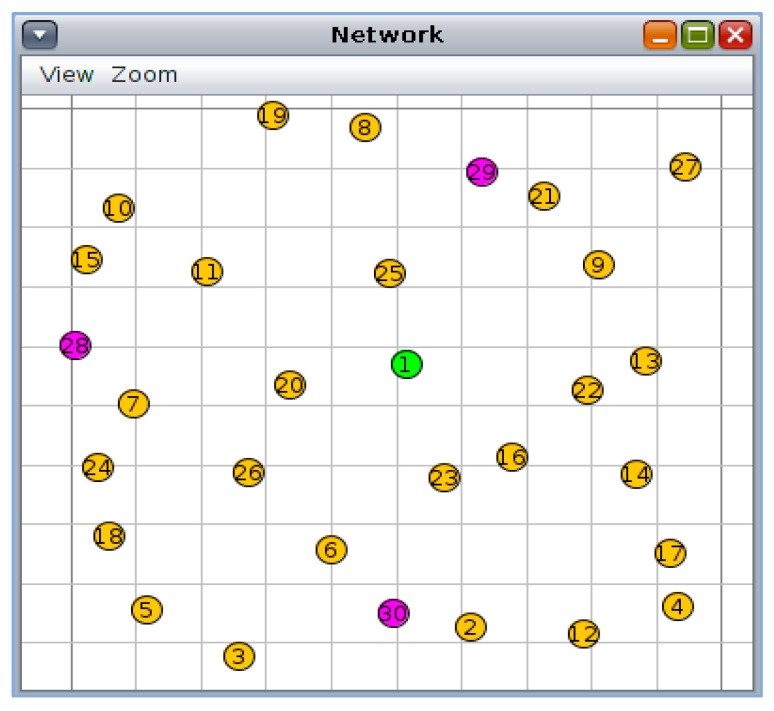
RPL Network of 30 nodes in Cooja simulator: sink node (1), sender nodes (2–27), attacker nodes (28, 29, 30).

**Figure 7:**
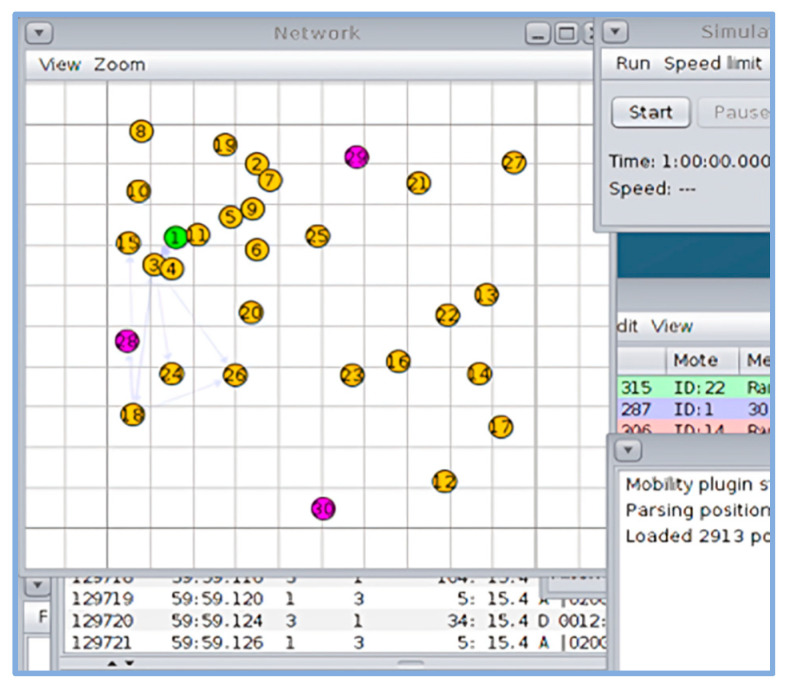
Network screenshot after 60 min simulation run, with mobile sink and Sender nodes in the network.

To evaluate the performance of SMTrust in a mobile IoT environment, a mobility plugin with Cooja is used. BonnMotion-3.0.1 tool [54] was used to generate the mobility scenario for nodes using the Random Waypoint mobility model. For a mobile IoT environment, mobility speed was kept as 0 to 6.23 km/h and mobile to static node ratio as 1:3 approximately [16]. Figure 6 shows the distribution of normal and attacker nodes in the network simulated using Cooja, with 1 sink node (node id: 1), 26 sender nodes (node id: 2–27), and 3 attacker nodes (node id: 28, 29, 30). Similarly, Figure 7 illustrates the network after 60 min simulation run with mobile sink and sender nodes in the network.

## 5. Performance Parameters

The performance of the proposed SMTrustOF-based protocol is compared with existing protocols based on topology stability, packet loss rate, throughput, and power consumption. Multiple simulation runs under three different scenarios have been used to verify the results for network performance comparison of SMTrust with MRHOF [55], SecTrust [14], MRTS [15], and DCTM [16]. The scenarios include Scenario I: static nodes, Scenario II: mobile sender nodes, and Scenario III: mobile sender and the sink node.

### 5.1. Node Rank Changes

**Figure 8:**
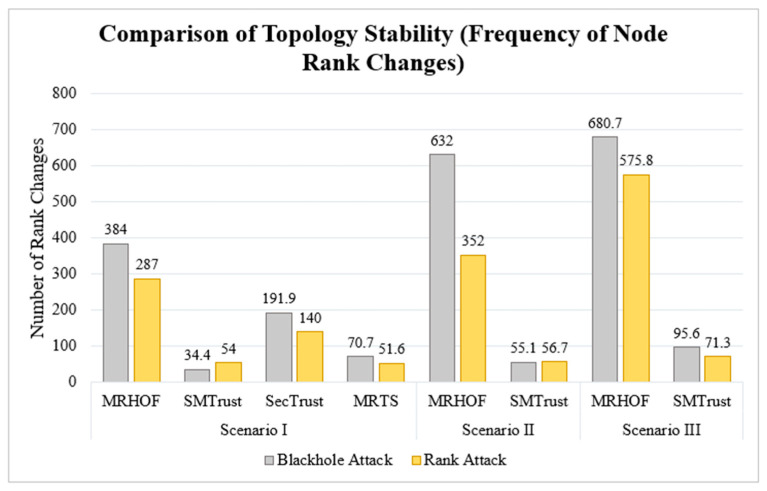
Comparison of the Node Rank Changes (frequency).

The frequency of rank change in the network indicates the stability of the topology, and hence the network. For MRHOF, the frequency of node rank change under both attacks is high in all three scenarios. To maintain the topology stability caused by the launching of attacks, the nodes tend to change parents frequently, thus causing a higher frequency of rank changes. The increased frequency of node rank changes in MRHOF (Figure 8) indicates an increased vulnerability to attacks, whereas SMTrustOF is marginally vulnerable. Though SecTrust outperforms MRHOF, SMTrust shows better results indicating more network and topology stability. Overall, SMTrust shows a smaller average frequency of node rank changes, indicating a more stable topology.

### 5.2. Packet Loss Rate

**Figure 9:**
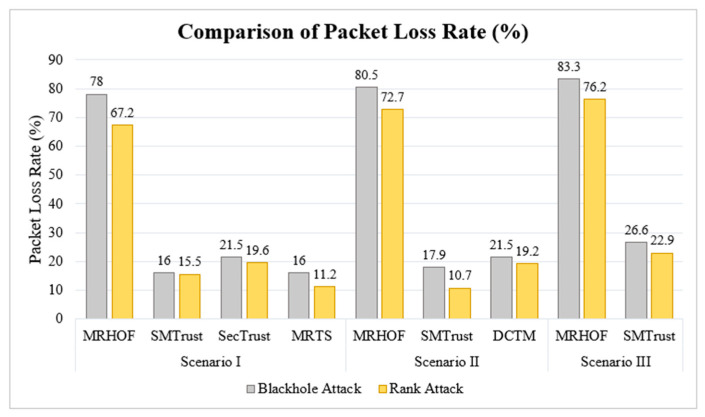
Comparison of Packet Loss Rate (%).

The packet collision and network congestion become disastrous under Blackhole and Rank attacks. The packet loss rate (PLR) for MRHOF in Scenario I is 78% and 67.2%, in Scenario II is 80.5% and 72.7%, and in Scenario III is 83.3% and 76.2%, under Blackhole and Rank attacks, respectively, as depicted in Figure 9. The graphical representation shows that the packet loss percentage under Blackhole and Rank attacks is much less in SMTrustOF as compared to MRHOF in all three scenarios. The higher PLR in MRHOF is since there is no implementation for detection of attacks or trust among nodes. In addition to that, there can be several causes, including that the normal nodes may choose a malicious parent that intends to drop the packets, thus disrupting the topology. Whereas, in SMTrustOF, the route is established considering trust metrics such as, success rate, historical observation, feedback, and energy level, thus reducing the overall packet loss rate.

Similarly, as compared to DCTM, SMTrust shows better results in Scenario II (Figure 9) with only 17.9% and 10.7% PLR, under Blackhole and Rank attack, respectively. The logic behind better results of SMTrust as compared to DCTM is the difference in the trust metrics and computation. Moreover, in contrast to DCTM, SMTrust uses location and link stability to compute the trustworthy route, and mobility of nodes is necessary to be considered when sender nodes are mobile in an IoT environment. The better results of SMTrust thus, indicate the importance of mobility metrics for trustworthy parent selection in an IoT scenario of mobile sender nodes.

For Scenario I, comprising static nodes, SMTrust also shows better results for PLR than SecTrust. This is because SMTrust has reduced node rank change frequency compared with SecTrust, thus causing a more stable topology and consequently reduced PLR. Moreover, the trust metrics and their computation in SMTrust is different as compared to SecTrust. Contrarily, SMTrust shows comparably acceptable results with MRTS. In MRTS, an IDS-based trust computation has been used which is computationally intensive in IoT scenarios and need an additional hardware module. Whereas, in comparison with MRTS, we show that keeping the trust computation simpler and selecting the crucial trust metrics for trust computation can improve the overall routing mechanism, particularly resulting in the reduced packet loss rate. Thus, indicating that the security from Blackhole and Rank attacks as well as a stable network topology can be achieved by carefully selecting the trust metrics instead of using computationally intensive IDS or hardware module.

### 5.3. Throughput

**Figure 10:**
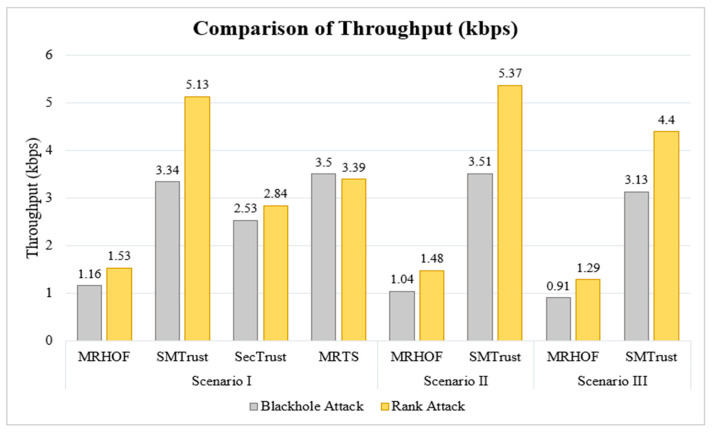
Comparison of Throughput (kbps).

The throughput of MRHOF is reduced drastically under attacks for Scenario I, as observed from Figure 10. This is because nodes select the malicious nodes as parents, and the affected normal nodes have a throughput of 0kbps because their packets do not reach the sink node. This shows that a part of the network is paralyzed for sending packets. Moreover, in MRHOF, there is no defence mechanism against attacks, and route is stablished without considering any of the trust metrics in contrast with the trustworthy route establishment in SMTrust, thus enhancing the throughput in the SMTrust-based RPL protocol. SMTrust, SecTrust, and MRTS offer higher throughput because of the embedded attack detection mechanisms. In addition, the nodes’ throughput remains greater than zero, thus increasing the throughput value overall. The average throughput value of SMTrust outperforms SecTrust in Scenario I, with a percentage increase of 32% and 80.6% under Blackhole and Rank attacks, respectively. The logic behind improved results of SMTrust is that it employs six different trust metrics as compared to other works, such as, success rate, recommended trust, historical observations, energy level, and location and link stability. Moreover, the difference in the quantification, and weighted computation of the overall trust index, as explained in Section 3, logically justifies its better performance.

In comparison with MRTS, SMTrust shows negligibly lesser throughput, a difference of 0.16 kbps, under Blackhole attack, and a higher packet loss rate, a difference of 4.3%, under Rank attack. This is because MRTS uses an IDS approach for attacks detection and isolation, as well as a hardware security chip embedded in each node. This is justified by the fact that SMTrust employs simplified equations to compute trust metrics and trust index, thus avoiding integration of IDS or hardware separately. This means that there is no requirement of hardware for embedding SMTrust in an IoT system. Similarly, SMTrust significantly outperforms MRHOF under attacks in Scenario II and III, as shown in Figure 10. Thus, indicating the importance of trust model and crucial selection of trust metrics for trustworthy topology creation and defence against routing attacks.

### 5.4. Power Consumption

**Figure 11:**
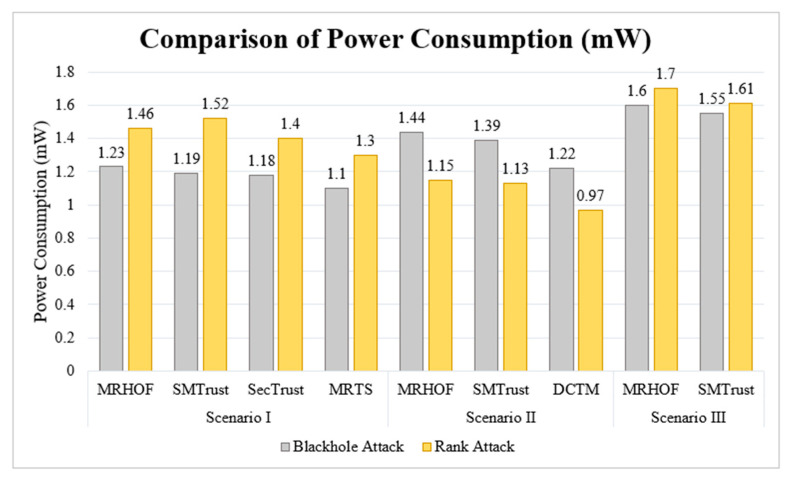
Comparison of Power Consumption (mW).

The average power consumption of SMTrustOF and MRHOF shows a small difference, which is justified as nodes in SMTrustOF perform trust computations. Though SMTrustOF shows lesser power consumption for all three scenarios under Blackhole attack, and for Scenario II and III under Rank attack. The power consumption of SMTrust is slightly higher as compared to MRHOF (under Rank attack in Scenario I), SecTrust, DCTM, and MRTS, as shown in Figure 11. However, the difference is negligibly small, that is, up to 0.22 mW. This is due to difference in computations involved for trust index, attack detection and trusted parent selection.

The power consumption in SMTrust is more because of the computations involved for trust metrics, and the DIO transmissions along with attack detection mechanism. Once the attacking nodes are detected and isolated by SMTrust, the overall power consumption of the network becomes stable. Furthermore, it is to be noted that the improvement of power consumption is beyond the scope for this research work; however, SMTrust shows a comparatively acceptable increase in power consumption.

Overall SMTrust demonstrates better performance for both Rank and Blackhole attacks. The performance of SMTrust is better for Scenario II as compared to Scenario I and Scenario III, indicating the importance of mobility-based trust metrics computation for parent selection algorithm. Moreover, the effects of sink mobility degrade the network performance for SMTrust under Rank and Blackhole attacks as compared to the results of Scenario I and II. However, SMTrust still outperforms in overall network performance parameters, including topology stability, PLR, and throughput measurement of nodes, indicating significant improvement as compared to existing systems.

## 6. Discussion

The exponential increase in the number of smart devices emphasize the need for security solutions in IoT domain. In this research, the security of RPL is focused, particularly the defense against Rank and Blackhole attacks. The trust-based approach is chosen because of its being lightweight [56], and viability of easy integration and implementation in IoT. Based on the critically chosen trust metrics, including the mobility-based metrics, a trust model is designed, and successfully integrated with RPL routing operation. The simulation study is carried out using ContikiOS/Cooja simulator for the performance evaluation of the proposed trust-based model for secure routing. The proposed SMTrust model outperforms the existing approaches. It provides security against RPL Rank and Blackhole attacks with enhanced network performance in terms of topology stability, throughput, packet loss rate, and power consumption.

The novelty and usefulness of the proposed trust-based model, in general, are summarized as follows:

- It provides secure communication in terms of routing among the resource-constrained nodes in IoT.
- It is suitable to be integrated into a P2P distributed network that consists of resource-constrained IoT nodes.
- It enhances secure, reliable, and trustworthy communication in IoT.
- It is a step closer to ensuring the availability and integrity of packet exchange in the network.
- It offers scalability with mobility of nodes and flexibility for various attacks detection and mitigation in RPL.

Overall, the performance of SMTrust is significantly better as compared to default RPL objective function MRHOF and existing trust-based approaches, such as SecTrust [14], DCTM [16], and MRTS [15].

## 7. Conclusions and Future Work

The IoT is emerging in several applications, such as, healthcare, grids, industries, traffic, etc. The number of IoT is increasing exponentially and is estimated to reach billions in coming years. The smart appliances usually are resource constrained; thus, a computationally intensive security mechanism cannot be embedded in the IoT. Several security solutions, including trust-based approaches, deep learning, and IDS, are proposed for networks security in the IoT. A trust-based method is suitable according to the limitations and requirements of the IoT. In existing literature, the mobility of nodes has been insufficiently addressed by trust-based approaches. Hence, it is imperative to explore trust-based security methods with optimum resource utilization for RPL routing attacks. This will help with IoT adoption on a wide scale. This research critically analyzes the parameters involved in trust computation and proposes security, mobility, and trust-based routing protocol, SMTrust. The proposed protocol allows only the trustworthy nodes to be selected as preferred parents and participate in the network. The proposed trust model has successfully outperformed MRHOF, SecTrust, DCTM, and MRTS. SMTrust shows enhanced performance in Scenario I, II and III, comprising static nodes, mobile sender nodes and mobile sink nodes, respectively. Thus, indicating the importance of mobility metrics for trust computation in mitigating RPL attacks in mobile IoT environment.

In the future, we plan to improve the power consumption and evaluate the proposed protocol for E2E delay and colluding attacks. Furthermore, the testbed implementation and experiments will be carried out accordingly.

## Figures and Tables

**Table 1:** Summary of the Recent Literature.

Ref	Technique(s) and Description	Attacks Considered	Mobility	Research Gaps
[14]	Using the fuzzy logic-based approach for threshold-based trust	Rank; Sybil	✘	Lack of node mobility, consideration for recommendation uncertainty, evaluation for colluding attacks, energy consumption, and E2E delays; Packet loss rate is significant.
[16]	A dynamic, comprehensive, multidimensional, hierarchical trust model.	Blackhole; Sybil; Rank	✓	Computing power can be improved; Sink node mobility and its impact on network performance is not considered
[29]	A feedback-aware trust-based protocol.	Blackhole; Selective forward	✘	Lack of node mobility, consideration for recommendation uncertainty, and evaluation for E2E delays, energy consumption, and colluding attacks; Packet loss rate is significant.
[15]	Introduces ETX as a metric for calculating trust in order to build a secure routing topology.	Rank; Blackhole	✘	Uses IDS-based attacks detection, and hardware security chip with each node; Lack of mobility of nodes.
[41]	Collaborative context parameters, trust degradation component, and recommendations.	On-Off attacks; Opportunistic service attacks;	✘	Not suitable for routing protocols.
[42]	Securing energy-efficient sensor network, recognizing the challenges of medical IoT mobility.	Greyhole	✓	Does not consider routing attacks and security.
[24]	Deep Learning based model	Hello Flooding Attacks	✘	Considered only the attacks against resources; Scalability problem; does not consider RPL attacks.
[31]	Trust-based PCC-RPL (Parental Change Control RPL)	Fabricated parent change	✘	Overhead due to IDS-based approach; No mitigation mechanism
[32]	Authentication and Trust-based IoT security with mobile sink.	Rank; Sybil; Blackhole	✓	Additional registration process for authentication of nodes; Frequent death of IoT nodes is alleviated.
[33]	A lightweight countermeasure based on adaptively adjusted thresholds	DIS attack	✘	WSN-inherited attacks are not considered; Only DIS-based attacks are defended
[34]	Distributes the load between the several modes	DIS flooding attacks	✘	Targeting load balancing to avoid DIS flooding attacks; Network related attacks are not considered.

**Table 2:** Trust Metrics in State-of-the-Art IoT Routing and Networks.

Ref	Domain	Trust Evaluation/Calculation/Metrics
[14]	Routing attacks; RPL	Historical observation; Feedback; Successful and unsuccessful transaction
[16]	Routing attacks; RPL	Contextual information; QoS; QPC
[44]	Routing Attacks; Wireless Networks	Historical Observations; Indirect trust; Route trust; Contextual factors
[12]	WSNs/LEACH; Attacks; Healthcare	Data packets received, dropped, and forwarded
[45]	RPL; Routing Attacks	Direct and Indirect trust
[46]	RPL; LLNs; WSNs	Node behavior
[47]	AODV; Routing Security	Direct trust; Historical observation; Uncertainty; Bayesian probability
[42]	Medical IoT; Routing	Energy consumption; Node capacity
[15]	IDS-based; RPL Attacks	Recommended trust; Energy; Honesty; Selfishness; ETX
[48]	RPL Security	Event-based trust; Weighted trust; Nonce ID; Timestamp
[49]	RPL Security	Routing behavior; Contextual factors; non-cooperative game models and DST

**Table 3:** Trust Metrics Applied for SMTrust.

Trust Metrics	Description
Success rate (*TM_SR_)*	The ratio of number of packets forwarded by the number of packets received.
Energy Level *(TM_EL_)*	Amount of remaining energy level of the node.
Historical Observations *(TM(H_0_))*	Recent trust value calculated for the node.
Location and Link Stability *(TM_LLS_)*	Node’s location based on Received Signal Strength Indicator (RSSI) value.
Mobility *(TM_Mobility_)*	Distance moved from the previously noted position.
Recommended Trust *(TM_RT_)*	Trust recommendation by 1-hop neighbors.

**Table 4:** Simulation Parameters.

Parameters	Values
Simulation Tool	InstantContiki2.7/Cooja
Simulation coverage area	110 m × 110 m
Total number of nodes	30
Malicious nodes	3
Deployment of nodes	Random positioning
TX range	50 m
INT range	60 m
TX ratio	100%
RX ratio	30–100%
Routing Protocol	MRHOF, SMTrustOF
Network protocol	IPv6 based
Start-up Delay	5000 milliseconds
Radio Medium	UDGM Distance Loss
Simulation period	60 min
Environment	Static, Mobile

## Data Availability

Data will be provided on request.
